# Vulnerabilidad de la obesidad definida por el índice de masa corporal, perímetro abdominal y porcentaje de grasa corporal

**DOI:** 10.1016/j.aprim.2022.102523

**Published:** 2022-12-27

**Authors:** Ricardo Ortega, Gonzalo Grandes, Sagrario Gómez-Cantarino, en nombre del Grupo PEPAF

**Affiliations:** a Centro de Salud de Santa Bárbara, Servicio de Salud de Castilla-La Mancha, Toledo, España; b Unidad de Investigación en Atención Primaria de Bizkaia, Servicio Vasco de Salud (Otsakidetza), Bilbao, España; c Departamento de Enfermería, Fisioterapia y Terapia Ocupacional, Facultad de Fisioterapia y Enfermería, Universidad de Castilla-La Mancha, Toledo, España

**Keywords:** Obesidad abdominal, Índice de masa corporal, Obesidad, Atención primaria, Pliegues grasos cutáneos, Perímetro abdominal, Abdominal obesity, Body mass index, Obesity, Primary health care, Skinfold thickness, Waist circumference

## Abstract

**Objetivo:**

Averiguar en qué medida es posible dejar de tener obesidad (normalizar el índice de masa corporal [IMC], el perímetro abdominal [PA] y/o el porcentaje de grasa corporal [PGC]).

**Diseño:**

Estudio de observación longitudinal y retrospectiva.

**Emplazamiento:**

Once centros de salud españoles.

**Participantes:**

Hombres y mujeres con IMC ≥ 30 kg/m^2^ (n = 1.246) u obesidad general (OG), con PA > 102 cm y > 88 cm, respectivamente (n = 2.122) u obesidad abdominal (OA) y con PGC > 25% y > 35%, respectivamente (n = 2.436) o exceso de grasa corporal (EGC), de la cohorte del Estudio PEPAF de 4.927 participantes de 20 a 80 años de edad.

**Mediciones principales:**

Datos procedentes del Estudio PEPAF de la captación y de 6, 12 y 24 meses: sexo, edad, diagnósticos de diabetes, hipertensión arterial y dislipemia, hábito tabáquico, niveles y cumplimiento de las recomendaciones de actividad física, consumo máximo de oxígeno, peso, talla, PA y tres pliegues grasos cutáneos (torácico, umbilical y muslo anterior para hombres y tríceps, suprailíaco y muslo anterior para mujeres).

**Resultados:**

De 2.054 participantes con cualquier tipo de obesidad en la captación y datos válidos a los 2 años, 240 (11,6%) habían normalizado todos sus índices diagnósticos de obesidad en ese tiempo. El 19,5% (intervalo de confianza al 95% (IC 95%): 17,6-21,4) habían dejado de tener EGC, el 12,0% (IC 95%: 10,4-13,7) habían dejado de tener OA y el 10,5% (IC 95%: 8,5-12,7) habían dejado de tener OG.

**Conclusiones:**

La obesidad se diferencia de las demás enfermedades crónicas en que es posible «curarse» de ella normalizando la cantidad de grasa corporal.

## Abstract (en)

**Aim:**

To ascertain to what extent it is possible to stop being obese (to normalize body mass index [BMI], waist circumference [WC] and/or body fat percentage [BFP]).

**Design:**

Longitudinal observational and retrospective study.

**Site:**

Eleven Spanish health centers.

**Participants:**

Men and women with BMI ≥ 30 kg/m^2^ (*n* = 1246) or general obesity (GO), with WC > 102 cm and > 88 cm, respectively (*n* = 2122) or abdominal obesity (AO) and with BFP > 25% and > 35%, respectively (*n* = 2436) or excess body fat (EBF), from the PEPAF Study cohort of 4927 participants aged 20-80 years.

**Main measurements:**

Data from the PEPAF study at baseline and at 6, 12 and 24 months: gender, age, diagnoses of diabetes, hypertension and dyslipidemia, smoking, levels of and compliance with physical activity recommendations, maximum oxygen consumption, weigh, height, WC and three skin-folds (thoracic, umbilical and anterior thigh for men and triceps, suprailiac and anterior thigh for women).

**Results:**

Of 2054 participants with any type of obesity at baseline and valid data at 2 years, 240 (11.6%) had normalized all of their obesity diagnostic indexes. 19.5% (95% confidence interval (95% CI: 17.6-21.4) ceased to have EBF, 12.0% (95% CI: 10.4-13.7) ceased to have AO and 10.5% (95% CI: 8.5-12.7) ceased to have GO.

**Conclusions:**

Obesity differs from other chronic diseases in that it can be «cured» by normalizing the amount of body fat.

## Introducción

La obesidad, entendida como un exceso de grasa corporal (EGC), se considera una enfermedad crónica similar a la hipertensión arterial, la diabetes o la dislipemia^1^, ^2^, ^3^, y cuando se acumula en el abdomen en forma de grasa visceral y subcutánea se considera obesidad abdominal (OA)^4^. Las enfermedades crónicas no se curan, solo se controlan con el tratamiento. Pero, en la obesidad, una persona puede perder ese exceso de grasa y convertirse en una persona con grasa normal a la que considerar sin obesidad y, por tanto, su enfermedad habrá desaparecido.

La acumulación de la grasa corporal en exceso se debe a un balance energético positivo del organismo, en el que las calorías que se ingieren con la alimentación (ingesta energética) superan a las calorías gastadas en todas las actividades que la persona realiza en 24 horas, incluido el sueño (gasto calórico)^5^. Esas calorías ingeridas que no se gastan se almacenan en forma de grasa.

La ingesta energética y el gasto calórico constituyen hábitos de vida presentes en todas las personas y en todo momento. Por eso es posible que, igual que han generado un balance energético positivo que les ha producido obesidad, generen después un balance negativo que les produzca la desaparición de esa obesidad.

Para diagnosticar obesidad en las consultas de atención primaria se utilizan: el índice de masa corporal (IMC)^1^, ^2^, ^3^, el perímetro abdominal (PA)^4^ y el porcentaje de grasa corporal (PGC)^6^.

Prácticamente todos los estudios sobre las intervenciones en obesidad pretenden valorar la reducción del peso y/o la grasa que esas intervenciones producen^7^, pero no buscan la proporción de personas obesas capaces de dejar de serlo.

El objetivo del presente estudio es cuantificar la proporción de pacientes de atención primaria que son capaces de normalizar el IMC, el PA o el PGC al cabo de 2 años, así como los factores que se asocian con la probabilidad de dejar de tener obesidad.

## Material y métodos

Este estudio se ha realizado con datos secundarios del estudio PEPAF, que no fueron analizados en el estudio original, en forma de estudio de observación longitudinal y retrospectiva. El estudio PEPAF era un ensayo clínico realizado en 11 centros de salud españoles, entre 2003 y 2006, con distribución aleatoria de los centros a un grupo de intervención o a un grupo control. Este estudio evaluó la efectividad de un Programa Experimental de Promoción de la Actividad Física para aumentar los niveles de actividad física (AF) de la población sedentaria de 20 a 80 años consultante en atención primaria. Para ello se reclutó una muestra de pacientes sin enfermedad cardiovascular conocida y sedentarios de acuerdo a un algoritmo, practicándose las mediciones del estudio a 4.927 participantes que cumplían esas condiciones. Posteriormente se les pasó a todos los participantes el *Physical Activity Readiness Questionnaire* (PAR-Q)^8^ para asegurar su sedentarismo, y se identificaron 850 participantes físicamente activos que fueron excluidos del ensayo clínico, cuya cohorte para dicho ensayo quedó establecida en 4.317 participantes. Estos participantes firmaron un consentimiento informado y se les siguió durante 2 años con mediciones en la captación y a los 6, 12 y 24 meses^9^, ^10^, ^11^.

A los participantes de intervención se les clasificó en preparados para modificar su nivel de AF y no preparados, de acuerdo con una versión reducida del modelo transteórico de las etapas de cambio^12^. A ambos grupos se les proporcionó una recomendación médica asertiva personalizada, de acuerdo con las evidencias disponibles sobre los beneficios del ejercicio y los riesgos de la inactividad. Los participantes no preparados recibieron un folleto centrado en la modificación de creencias sobre la AF, y de forma oportuna volvieron a ser abordados en consultas posteriores para conocer su intención de modificar su hábito sedentario. A los participantes preparados se les citó para realizar una consulta adicional de menos de 20 minutos de duración para abordar las posibles barreras anticipadas por el participante (en cuanto a falta de tiempo, recursos o problemas de salud), y se negoció con ellos un Plan de Actividad Física (PAF) centrado en el cumplimiento y superación de las recomendaciones de los *Centers for Disease Control and Prevention*^13^. El PAF se entregó al participante a modo de prescripción y se evaluó de forma oportunista en las siguientes consultas.

El Estudio PEPAF cumplía las directrices de la Declaración de Helsinki y su protocolo^9^ fue aprobado por los Comités Éticos de Investigación Clínica (CEIC) de los centros participantes (ClinicalTrials.gov Identifier: NCT00131079).

### Población

Para el presente estudio se eligieron, de entre los 4.927 participantes con mediciones basales, a los que tenían un IMC ≥ 30 kg/m^2^ (n = 1.246) para la obesidad general (OG)^2^, a los hombres y mujeres que tenían, respectivamente, un PA > 102 cm y > 88 cm (n = 2.122) para la OA^4^, ^14^ y a los hombres y mujeres que tenían, respectivamente, un PGC > 25% y > 35% (n = 2.436) para el EGC^6^, ^15^, ^16^.

### Mediciones

Se seleccionaron las siguientes variables sociodemográficas basales del Estudio PEPAF: sexo, edad y diagnósticos de diabetes, hipertensión arterial y dislipemia; y también hábito tabáquico, niveles de AF, cumplimiento de las recomendaciones de AF y mediciones de consumo máximo de oxígeno (VO_2_max), peso, talla, PA y tres pliegues grasos cutáneos (torácico, umbilical y muslo anterior para hombres, y tríceps, suprailíaco y muslo anterior para mujeres), correspondientes a las visitas de captación y a los 6, 12 y 24 meses.

El hábito tabáquico se obtuvo por autodeclaración, y se codificó como fumador y no fumador.

Se consideraron como en inactividad física los participantes que no cumplían las recomendaciones de AF (al menos 30 minutos de AF moderada 5 días a la semana, o 20 minutos de AF vigorosa 20 minutos a la semana, o combinaciones híbridas de episodios de AF moderada e intensa)^17^ según su AF habitual. La medición de la AF se hizo con el cuestionario PAR-Q^8^.

El VO_2_max se estimó de forma indirecta utilizando el protocolo YMCA-ACSM^18^ en una prueba de esfuerzo submáximo con un cicloergómetro VarioBike 500 y fue estandarizado para edad, sexo y frecuencia cardiaca de reposo.

El peso y la estatura se midieron en una báscula de consulta calibrada y con tallímetro, con el participante descalzo y con la ropa mínima, y manteniendo la cabeza en posición de alineamiento de la nariz y la oreja, y se calculó el IMC mediante la fórmula: peso (kg)/talla^2^ (m).

El PA se midió con el paciente tumbado en la camilla, con el abdomen descubierto, y rodeando el abdomen a nivel del ombligo con una cinta métrica plastificada.

Los pliegues grasos cutáneos se midieron siguiendo los protocolos estandarizados de Jackson y Pollock^19^, y con ellos se obtuvo la densidad corporal que se llevó a la fórmula de Siri^20^ para obtener el PGC.

La forma física cardiorrespiratoria (FFCR) de los participantes se categorizó en baja, media y alta según los tertiles de VO_2_max en cada sexo, considerando FFCR buena al tertil de alta y mala a los otros dos.

### Variables de resultados

Proporciones de participantes que habían normalizado su IMC (< 30 kg/m^2^), su PA (≤ 102 cm en hombres y ≤ 88 cm en mujeres) o su PGC (≤ 25% en hombres y ≤ 35% en mujeres) a los 2 años.

### Análisis estadístico

Se realizó con el paquete estadístico STATA. Se calcularon las medias ± desviaciones estándar para las variables cuantitativas y los porcentajes de participantes en cada categoría para las variables cualitativas. Los grupos se compararon utilizando el test de chi cuadrado para las proporciones y el test de Student o el análisis de la varianza para las medias ± desviaciones estándar. La asociación de las proporciones de los que dejaron de tener cada una de las tres obesidades con el resto de variables del estudio se computó como la odds ratio (OR) de cada una de las variables en las proporciones de los que habían dejado de tener cada obesidad a los 2 años divididas por la OR de las proporciones de los participantes que seguían teniendo esas obesidades. Se utilizaron modelos mixtos de regresión logística multivariantes para el ajuste de potenciales variables de confusión y para obtener las odd ratios ajustadas (ORA).

**Figure:**
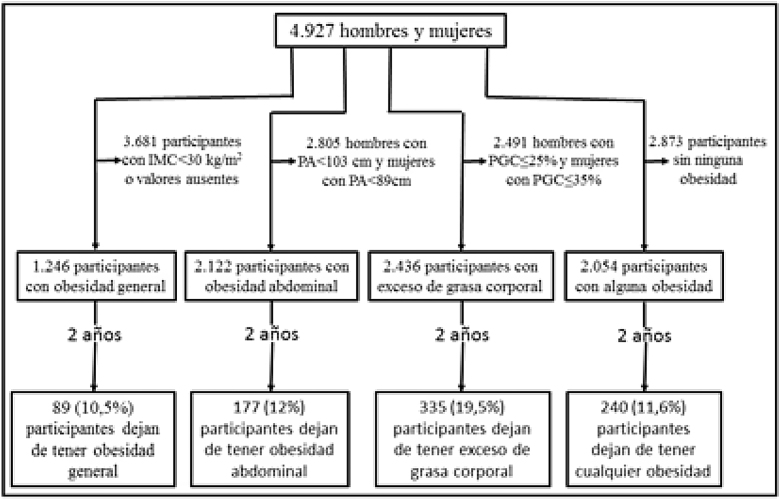
**Esquema general del estudio**.

## Resultados

La tabla 1 presenta las características principales de las muestras. Se observa que la proporción de OG en los participantes con PA elevado (52,9%) es mayor (p < 0,001) que en los que tienen PGC elevado (44,5%); la proporción de OA en los participantes con IMC elevado (90,4%) es mayor (p < 0,001) que en los que tienen PGC elevado (69,7%), y la proporción de EGC en los participantes con IMC elevado (87,0%) es mayor (p < 0,001) que en los que tienen PA elevado (79,8%).

**Tabla 1:** Características de las muestras de los tres tipos de obesidad en la captación, en total y distribuidos por sexo

	Obesidad general IMC elevado			Obesidad abdominal PA elevado			Exceso grasa corporal PGC elevado^c^		
	Total n = 1.246	♂ n = 418	♀ n = 828	Total n = 2.122	♂ n = 474	♀ n = 1.648	Total n = 2.436	♂ n = 854	♀ n = 1.582
*Edad, años*	55,0 ± 13,4	53,5 ± 14,0	55,8 ± 13,0	55,6 ± 13,1	56,1 ± 13,2	55,4 ± 13,1	54,6 ± 13,3	54,7 ± 13,9	54,5 ± 13,0
*Peso, kg*	86,1 ± 12,4	93,8 ± 11,2	82,1 ± 11,1	78,6 ± 13,9	92,6 ± 11,3	74,5 ± 11,8	78,2 ± 13,8	85,8 ± 12,1	74,0 ± 12,1
*IMC, kg/m*^*2*^	33,4 ± 3,2	32,6 ± 2,5	33,8 ± 3,4	30,7 ± 4,2	31,7 ± 3,1	30,4 ± 4,4	30,0 ± 4,1	29,7 ± 3,4	30,2 ± 4,5
*PA, cm*	106,2 ± 10,1	108,4 ± 9,0	105,1 ± 10,4	102,4 ± 9,8	109,4 ± 7,6	100,4 ± 9,5	100,1 ± 11,0	102,5 ± 9,4	98,7 ± 11,5
*PGC, %*	36,8 ± 6,6	29,4 ± 3,8	40,5 ± 4,2	36,5 ± 5,9	29,1 ± 4,0	38,7 ± 4,4	36,1 ± 6,0	29,1 ± 2,9	39,9 ± 3,1
*VO*_*2*_*max, ml/kg/min*	21,5 ± 7,3	27,0 ± 7,4	18,6 ± 5,3	20,9 ± 6,4	26,3 ± 7,1	19,3 ± 5,3	22,4 ± 7,3	27,5 ± 7,7	19,6 ± 5,2
*AF*									
MET-h/s	0,62 ± 1,8	0,83 ± 2,5	0,51 ± 1,3	0,64 ± 1,4	0,79 ± 1,7	0,60 ± 1,3	0,70 ± 1,8	0,87 ± 2,3	0,61 ± 1,4
Min/sem	72 ± 224	107 ± 331	54 ± 138	69 ± 168	92 ± 233	63 ± 144	75 ± 200	96 ± 268	63 ± 150
*Fumadores*	20,8 (18,6-23,2)	31,3 (26,9-36,0)	15,5 (13,1-18,2)	16,5 (14,7-18,3)	32,9 (28,6-37,3)	9,4 (7,5-11,6)	22,7 (21,0-24,4)	31,8 (28,7-35,0)	17,7 (15,9-19,7)
*No cumple RAF*	90,0 (88,2-91,6)	88,9 (85,5-91,8)	90,5 (88,3-92,4)	88,9 (87,5-90,3)	88,1 (84,9-90,9)	89,2 (87,6-90,6)	88,9 (87,6-90,1)	88,2 (85,9-90,3)	89,2 (87,6-90,7)
*FFCR mala*	83,4 (80,8-85,7)	80,8 (76,1-85,0)	84,8 (81,7-87,6)	81,6 (79,6-83,5)	82,5 (78,2-86,3)	81,3 (79,1-83,4)	79,8 (78,0-81,6)	77,3 (73,9-80,4)	81,3 (79,0-83,4)
*DM2*	15,3 (13,3-17,4)	17,9 (14,3-21,9)	14,0 (11,7-16,5)	13,0 (11,6-14,5)	19,1 (15,7-23,0)	11,2 (9,7-12,8)	11,0 (9,8-12,3)	14,0 (11,7-16,5)	9,4 (8,0-11,0)
*HTA*	45,5 (42,7-48,3)	44,9 (40,1-49,8)	45,7 (42,3-49,2)	38,3 (36,2-40,4)	48,1 (43,5-52,7)	35,5 (33,2-37,9)	34,7 (32,8-36,6)	37,3 (34,0-40,6)	33,3 (31,0-35,7)
*Dislipemia*	27,2 (24,7-29,7)	28,2 (23,9-32,8)	26,6 (23,7-29,8)	26,9 (25,0-28,8)	27,8 (23,8-32,1)	26,6 (24,5-28,8)	25,6 (23,9-27,4)	26,9 (23,9-30,0)	25,0 (22,9-27,2)
*OG*	×	×	×	52,9 (50,7-55,0)	69,4 (65,0-73,5)	48,1 (45,7-50,6)	44,5 (42,5-46,5)	42,0 (38,7-45,4)	45,8 (43,4-48,3)
*OA*	90,4 (88,6-91,9)	78,8 (74,6-82,7)	96,2 (94,7-97,4)	×	×	×	69,7 (67,8-71,5)	46,3 (42,9-49,7)	82,3 (80,3-84,2)
*EGC*	87,0 (85,0-88,8)	85,8 (82,1-89,0)	87,6 (85,2-89,8)	79,8 (78,0-81,5)	83,3 (79,6-86,5)	78,8 (76,7-80,7)	×	×	×

AF: actividad física; DM2: diabetes mellitus tipo 2; EGC: exceso de grasa corporal, FFCR: forma física cardiorrespiratoria; HTA: hipertensión arterial; IMC: índice de masa corporal ≥ 30 kg/m^2^; MET-h/s: unidades metabólicas por hora a la semana; Min/sem: minutos a la semana; OA: obesidad abdominal; OG: obesidad general; PA: perímetro abdominal > 102 cm en hombres y 88 cm en mujeres; PGC: porcentaje de grasa corporal > 25% en hombres y > 35% en mujeres; RAF: recomendaciones de actividad física; VO_2_max: consumo máximo de oxígeno.

Variables edad, peso, IMC, PA, PGC, VO2max y AF expresadas en media ± desviación estándar. Resto de variables expresadas como % (intervalo de confianza al 95%).

La tabla 2 muestra la proporción de los que dejan de tener OG, OA o EGC a los 2 años, siendo significativamente (p < 0,05) más alta la proporción de EGC que las de OG u OA. También muestra que la proporción de hombres que dejan de tener OA es significativamente mayor que la de las mujeres (p = 0,001).

**Tabla 2:** Proporción de participantes que dejan de tener cada una de las tres obesidades y comparación entre hombres y mujeres a los 2 años

	Total		Hombres		Mujeres		
	n	% (IC 95%)	n	% (IC 95%)	n	% (IC 95%)	p
OG	89	10,5 (8,5-12,7)	40	13,2 (9,6-17,4)	49	9,0 (6,7-11,6)	0,056
OA	177	12,0 (10,4-13,7)	59	17,3 (13,4-21,7)	118	10,4 (8,7-12,3)	0,001
EGC	335	19,5 (17,6-21,4)	133	21,1 (17,9-24,5)	202	18,6 (16,3-21,0)	0,209

EGC: exceso de grasa corporal; IC 95%: intervalo de confianza al 95%; n: número; OA: obesidad abdominal; OG: obesidad general; p: significación; %: porcentaje.

De 2.054 participantes con uno o más tipos de obesidad en la captación y datos válidos a los 2 años, 240 (11,6%) participantes (108 [15,2%] hombres y 132 [9,8%] mujeres) habían normalizado todos sus índices diagnósticos de obesidad en ese tiempo.

A los 2 años: de 89 participantes con IMC normal, 30 (33,7%; IC 95%: 24,0-44,5) lo habían normalizado antes de los 6 meses; de 177 participantes con PA normal, 59 (33,3%; IC 95%: 26,6-40,7) lo habían normalizado antes de los 6 meses, y de 335 participantes con PGC normal, 103 (30,7%; IC 95%: 25,8-35,9) lo habían normalizado antes de los 6 meses.

En la tabla 3 se observa que: son mayores los aumentos en los niveles de AF en MET-h/semana (p < 0,001) y minutos/semana (p < 0,03) en los que dejan de tener OG y el aumento del VO_2_max (p < 0,03) en los que dejan de tener OA o EGC que en los que siguen con esas obesidades; los que dejan de tener OG son los que más reducen su peso y su IMC (6,8 kg y 2,6 kg/m^2^ frente a 2,5 y 0,9 de la OA y 2,8 y 1 del EGC), así como los que más aumentan sus niveles de AF (0,9 MET-h/semana y 60 minutos/semana frente a 0,3 y 0,2 MET-h/semana y 20 y 8 minutos/semana de la OA y el EGC, respectivamente); los que dejan de tener OA son los que más PA pierden (8,2 cm frente a 6,5 y 3,4, respectivamente, de la OG y el EGC); y los que dejan de tener EGC son los que más PGC pierden (4,1% frente al 2,8% y al 2,4%, respectivamente, de la OG y la OA) y los que más aumentan su VO_2_max (1,7 frente a 1,2 y 1,0 ml/kg/min, respectivamente, de la OG y la OA).

**Tabla 3:** Cambios en las variables cuantitativas de los que dejan de tener cada una de las tres obesidades y los que siguen con ellas y su comparación a los 2 años

	Obesidad general					Obesidad abdominal					Exceso grasa corporal				
	No		Sí			No		Sí			No		Sí		
	n	x ± DE	n	x ± DE	p	n	x ± DE	n	x ± DE	p	n	x ± DE	n	x ± DE	p
*Peso, kg*	89	−6,4 ± 5,2	761	0,4 ± 4,2	< 0,001	177	−2,2 ± 4,7	1.296	0,3 ± 4,0	< 0,001	335	−2,4 ± 4,8	1.379	0,4 ± 3,8	< 0,001
*IMC, kg/m*^*2*^	89	−2,5 ± 1,9	761	0,1 ± 1,6	< 0,001	177	−0,8 ± 1,7	1.296	0,1 ± 1,6	< 0,001	335	−0,9 ± 1,8	1.379	0,1 ± 1,4	< 0,001
*PA, cm*	89	−5,8 ± 10,2	758	0,7 ± 6,1	< 0,001	177	−7,5 ± 7,4	1.297	0,7 ± 5,9	< 0,001	335	−2,8 ± 7,2	1.377	0,6 ± 5,9	< 0,001
*PGC, %*	89	−3,2 ± 4,0	760	-0,4 ± 3,0	< 0,001	177	−2,7 ± 3,4	1.297	−0,3 ± 3,3	< 0,001	335	−4,4 ± 2,8	1.380	−0,3 ± 2,7	< 0,001
*AF*															
MET-h/sem	89	1,7 ± 3,2	758	0,8 ± 2,3	< 0,001	177	1,1 ± 2,7	1.294	0,8 ± 2,3	0,101	335	1,1 ± 2,6	1.375	0,9 ± 2,4	0,119
Min/sem	89	136 ± 441	758	76 ± 250	0,026	177	102 ± 288	1.294	82 ± 245	0,155	335	98 ± 258	1.375	90 ± 251	0,299
*VO*_*2*_*max, ml/kg/min*	66	1,9 ± 6,0	450	0,7 ± 5,7	0,053	126	1,6 ± 5,4	795	0,6 ± 5,7	0,026	256	2,2 ± 6,1	886	0,5 ± 5,7	< 0,001

AF: actividad física; IMC: índice de masa corporal; MET-h/sem: unidades metabólicas por hora a la semana; Min/sem: minutos/semana; n: número; p: significación; PA: perímetro abdominal; PGC: porcentaje de grasa corporal; VO_2_max: consumo máximo de oxígeno. x ± DE: media ± desviación estándar.

Como muestra la tabla 4, a los 2 años: los que dejan de tener OA tienen una proporción mayor (p < 0,005) de los que siguen fumando y menor de los que siguen sin fumar; los que dejan de tener OG tienen una proporción mayor (p < 0,013) de los que dejan de cumplir las recomendaciones de AF y menor de los que siguen sin cumplirlas; y los que dejan de tener OG, OA o EGC tienen una proporción menor (p < 0,002) de los que mantienen una mala FFCR y mayor de los que la mantienen buena. Así mismo, los que dejan de tener OG tienen porcentajes de OA y de EGC menores (p < 0,001); los que dejan de tener OA tienen porcentajes de OG y de EGC menores (p < 0,001), y los que dejan de tener EGC tienen porcentajes de OA menores (p < 0,001).

**Tabla 4:** Cambios en las variables cualitativas de los que dejan de tener cada una de las tres obesidades y los que siguen con ellas y su comparación a los 2 años

	Obesidad general					Obesidad abdominal					Exceso grasa corporal				
	No		Sí			No		Sí			No		Sí		
	n	% (IC 95%)	n	% (IC 95%)	p	n	% (IC 95%)	n	% (IC 95%)	p	n	% (IC 95%)	n	% (IC 95%)	p
*Fumador*					0,126					0,004					0,064
Sí-Sí	16	17,9	102	13,4		38	21,4	167	12,8		68	20,2	206	14,9	
		(10,6-27,5)		(11,0-16,0)			(15,6-28,2)		(11,1-14,8)			(16,1-25,0)		(13,0-16,9)	
Sí-No	7	7,8	27	3,5		7	3,9	45	3,4		14	4,1	63	4,5	
		(3,2-15,5)		(2,3-5,1)			(1,6-7,9)		(2,5-4,6)			(2,3-6,9)		(3,5-5,8)	
No-Sí	1	1,1	12	1,5		6	3,3	21	1,6		7	2,0	18	1,3	
		(0,02-6,1)		(0,8-2,7)			(1,2-7,2)		(1,0-2,4)			(0,8-4,2)		(0,7-2,0)	
No-No	65	73,0	620	81,4		126	71,1	1064	82,0		246	73,4	1093	79,2	
		(62,5-81,8)		(78,5-84,1)			(63,9-77,7)		(79,8-84,0)			(68,3-78,0)		(76,9-81,3)	
*Cumple RAF*					0,012					0,053					0,540
No-No	49	55,0	516	68,0		105	59,3	846	65,3		200	59,7	876	63,7	
		(44,1-65,6)		(64,6-71,3)			(51,6-66,6)		(62,7-67,9)			(54,2-64,9)		(61,1-66,2)	
No-Sí	6	6,7	34	4,4		17	9,6	62	4,7		18	5,3	67	4,8	
		(2,5-14,0)		(3,1-6,2)			(5,6-14,9)		(3,6-6,1)			(3,2-8,3)		(3,7-6,1)	
Sí-No	31	34,8	159	20,9		43	24,2	303	23,4		88	26,2	335	24,3	
		(25,0-45,6)		(18,1-24,0)			(18,1-31,2)		(21,1-25,8)			(21,6-31,3)		(22,1-26,7)	
Sí-Sí	3	3,3	49	6,4		12	6,7	83	6,4		29	8,6	97	7,0	
		(0,7-9,5)		(4,8-8,4)			(3,5-11,5)		(5,1-7,8)			(5,8-12,1)		(5,7-8,5)	
*FFCR*					0,001					< 0,001					< 0,001
Mala-Mala	36	54,5	334	74,2		68	53,9	561	70,5		139	54,2	618	69,7	
		(41,8-66,8)		(69,9-78,2)			(44,8-62,8)		(67,2-73,7)			(47,9-60,5)		(66,6-72,7)	
Mala-Buena	5	7,5	39	8,6		12	9,5	67	8,4		14	5,4	77	8,6	
		(2,5-16,8)		(6,2-11,6)			(5,0-16,0)		(6,5-10,5)			(3,0-9,0)		(6,9-10,7)	
Buena-Mala	14	21,2	49	10,8		21	16,6	100	12,5		47	18,3	106	11,9	
		(12,1-33,0)		(8,1-14,1)			(10,6-24,3)		(10,3-15,0)			(13,8-23,6)		(9,8-14,2)	
Buena-Buena	11	16,6	28	6,2		25	19,8	67	8,4		56	21,8	85	9,5	
		(8,6-27,8)		(4,1-8,8)			(13,2-27,8)		(6,5-10,5)			(16,9-27,4)		(7,7-11,7)	
*OG*	×	×	×	×		36	20,3	741	57,1	< 0,001	57	17,0	683	49,5	< 0,001
							(14,6-27,0)		(54,4-59,8)			(13,1-21,4)		(46,8-52,2)	
*OA*	51	57,3	689	90,5	< 0,001	×	×	×	×		133	39,7	1006	72,8	< 0,001
		(46,3-67,7)		(88,2-92,5)								(34,4-45,1)		(70,4-75,2)	
*EGC*	53	59,5	645	84,8	0,053	84	21,4	1016	78,4	< 0,001	×	×	×	×	
		(48,6-69,8)		(82,1-87,3)			(15,6-28,2)		(76,1-80,6)						

EGC: exceso de grasa corporal; FFCR: forma física cardiorrespiratoria; IC 95%: intervalo de confianza al 95%; n: número; OA: obesidad abdominal; OG: obesidad general; p: significación; RAF: recomendaciones de actividad física; %, porcentaje.

Al analizar la relación de las obesidades entre sí, entre los que normalizan su IMC a los 2 años el 22% han normalizado también su PA más su PGC, el 20% solo su PA y el 18% solo su PGC, pero queda un 39% con un PA más un PGC elevados que no elevan el IMC. Entre los que normalizan su PA a los 2 años el 47% han normalizado también su IMC más PGC, el 33% solo su IMC y el 6% solo su PGC, pero queda un 15% con un PA normal y un PGC elevado que también elevan el IMC. Entre los que normalizan su PGC a los 2 años el 56% han normalizado también su IMC más su PA, el 27% solo su IMC y el 4% solo su PA, pero queda un 13% con un PGC normal y un PA elevado que también elevan el IMC.

En las Tabla 5, Tabla 6 se puede ver que: dejar de tener OG solo se asocia con ser mujer, tener hipertensión y el cambio en la FFCR; dejar de tener OA solo se asocia con la edad, el cumplimiento de las recomendaciones de AF en la captación y el cambio de ese cumplimiento y en la FFCR a los 2 años, y dejar de tener EGC solo se asocia con la edad y el cambio en la FFCR a los 2 años.

**Tabla 5:** Factores de la captación asociados con la probabilidad de dejar de tener alguna de las tres obesidades a los 2 años: odds ratios con ajuste multivariante

	ORA (IC 95%)^a^	p
*Obesidad general*		
Sexo: mujer vs. hombre	0,58 (0,35-0,98)	0,044
HTA: sí vs. no	0,46 (0,24-0,86)	0,015
*Obesidad abdominal*		
Edad	0,97 (0,95-0,98)	0,001
Cumple RAF: sí vs. no	1,70 (1,04-2,76)	< 0,001
*Exceso de grasa corporal*		
Edad	0,97 (0,96-0,99)	< 0,001

IC 95%: intervalo de confianza del 95%; ORA: odds ratio ajustada; p: significación; RAF: recomendaciones de actividad física.

a Ajustado para sexo, edad, niveles de actividad física, tabaco, cumplimiento de recomendaciones de actividad física, diabetes mellitus, hipertensión arterial, dislipemia y forma física cardiorrespiratoria.

**Tabla 6:** Asociaciones de cambio de hábitos con la probabilidad de dejar de tener alguna de las tres obesidades a los 2 años: odds ratios con ajuste multivariante

	ORA (IC 95%)^a^	p
**Obesidad general**		
*FFCR*		0,001
Mala-Buena vs. Mala-Mala	2,66 (1,31-5,41)	
Buena-Mala vs. Mala-Mala	1,35 (0,48-3,77)	
Buena-Buena vs. Mala-Mala	3,89 (1,73-8,78)	
**Obesidad abdominal**		
*Cumple RAF*		0,053
No-Sí vs. No-No	1,07 (0,67-1,69)	
Sí-No vs. No-No	2,49 (1,22-5,09)	
Sí-Sí vs. No-No	1,41 (0,69-2,86)	
*FFCR*		< 0,001
Mala-Buena vs. Mala-Mala	1,64 (0,95-2,84)	
Buena-Mala vs. Mala-Mala	1,42 (0,72-2,78)	
Buena-Buena vs. Mala-Mala	2,82 (1,64-4,84)	
**Exceso de grasa corporal**		
*FFCR*		< 0,001
Mala-Buena vs. Mala-Mala	1,99 (1,34-2,94)	
Buena-Mala vs. Mala-Mala	0,83 (0,45-1,52)	
Buena-Buena vs. Mala-Mala	2,99 (2,03-4,41)	

FFCR: forma física cardiorrespiratoria; IC 95%: intervalo de confianza del 95%; ORA: odds ratio ajustada; p: significación; RAF: recomendaciones de actividad física.

a Ajustado para cambios en: hábito tabáquico, cumplimiento de recomendaciones de actividad física y forma física cardiorrespiratoria.

## Discusión

Como muestran los resultados de este estudio, es posible dejar de tener cualquiera de las tres obesidades o las tres juntas; es decir, normalizar el IMC, el PA y/o el PGC a los 2 años. En concreto, el 11,6% de los participantes con uno o más tipos de obesidad en la captación pasan a ser normales en ese tiempo, y alrededor de un tercio lo hacen antes de los 6 meses. Y analizando las tres obesidades por separado, el 20% han dejado de tener EGC, el 12% OA y el 11% OG en ese tiempo. Luego, es posible dejar de tener la enfermedad de la obesidad y mantenerse sin ella.

Los resultados también indican que lo primero que disminuye es un conjunto de grasa general y abdominal subcutánea, o PGC, y después se une una disminución de grasa abdominal (visceral y/o subcutánea), o PA, que alcanzan en un momento dado un impacto en el peso corporal suficiente para reducir el IMC por debajo de 30 kg/m^2^.

De lo anterior se deriva que: puede ser más difícil normalizar el IMC, dado que para reducir el IMC hay que conseguir reducir a la vez el PA y el PGC en cifras superiores a los de otras obesidades; el EGC revierte más a los 2 años, posiblemente porque la acumulación excesiva de grasa abdominal constituye el 70% de la de grasa corporal total (de EGC), significando que la grasa que primero se pierde es la periférica, que suele ser más subcutánea que visceral, y después se pierde la grasa visceral del abdomen^21^; cuando la pérdida de grasa de los dos tipos alcanza una cantidad que supone un determinado peso corporal, se reduce el IMC y se deja de tener OG.

Cuando consideramos libres de obesidad a aquellos con IMC < 30 kg/m^2^, todavía hay una proporción considerable de OA y/o de EGC entre ellos, mientras que cuando consideramos libres de obesidad a aquellos con PA normal, la proporción de los que siguen con OG o EGC entre ellos es mucho menor.

Cada año de aumento en la edad supone un 3% menos probabilidad de dejar de tener OA o EGC a los 2 años, lo que significa que a mayor edad cuesta más trabajo perder grasa, porque con el envejecimiento va disminuyendo la masa muscular y aumentando la masa grasa^22^.

A los 2 años, pasar de una mala a buena FFCR desde la captación y mantenerla buena en ese tiempo se asocia, respectivamente, con una mayor probabilidad (del 166% y del 289%) de dejar de tener OG, y una mayor probabilidad (del 99% y del 199%) de dejar de tener EGC; y dejar de cumplir las recomendaciones sobre AF desde la captación y mantener una buena FFCR en ese tiempo se asocia, respectivamente, con una mayor probabilidad (del 149% y del 182%) de dejar de tener OA a los 2 años.

No hemos encontrado ningún estudio en que el objetivo sea la desaparición de la obesidad. Sin embargo, en un estudio americano sobre la incidencia de obesidad^23^ se informa que entre 1990 y 2000, después de 8 años de seguimiento, el 10,8% de sujetos con IMC ≥ 30 kg/m^2^ (11% de hombres y 10,7% de mujeres) reducían su IMC por debajo de esa cifra. Esos porcentajes son similares a los de este estudio, que sería el primer estudio en demostrar que la obesidad, como enfermedad, es reversible. Sí hay muchos estudios de intervención en la obesidad cuyo objetivo es disminuir el peso corporal^24^ o la grasa visceral^24^, ^25^ o general^24^, ^26^ en un determinado porcentaje o hasta un determinado límite.

### Limitaciones y fortalezas

Posibles limitaciones de este estudio son: la fecha de los datos recogidos (2003-2006), que haría que las cifras hayan cambiado, pero dan idea de las posibilidades de revertir la obesidad; y la posibilidad de que la pérdida de peso se debiera a una enfermedad crónica adelgazante, pero se puede argumentar que los participantes con esas enfermedades no acudieron a las mediciones o no se les realizaron.

Se podría señalar como limitación el hecho de que se trata de una muestra en la que una parte de ella ha sido intervenida con el PAF para aumentar sus niveles de AF. Pero ese hecho tiene el mismo impacto en los porcentajes que si el participante ha seguido un plan de reducción de dieta y/o de aumento de AF adoptado por sí mismo o prescrito por un profesional sanitario, porque para que una persona deje de ser obesa necesita obligatoriamente una intervención con dieta y/o ejercicio.

Como fortalezas se puede señalar el entrenamiento del personal investigador y el control de calidad llevado a cabo en el estudio PEPAF.

## Conclusiones

La obesidad como enfermedad crónica se diferencia de las demás en que es posible «curarse» de ella normalizando la cantidad de grasa corporal y manteniéndose así.

A los 2 años, lo que más se normaliza es el PGC y con ello el EGC, después el PA y con ello la OA, y por último el IMC y con ello la OG.

Entre los que normalizan su IMC, la mitad siguen teniendo OA o EGC, mientras que entre los que normalizan su PA, solo una quinta parte siguen teniendo OG o EGC.

## Consideraciones éticas

El protocolo del Estudio PEPAF fue aprobado por los comités éticos de investigación de todos los centros de salud participantes. Se solicitó el consentimiento informado a todos los participantes para su inclusión en el Estudio PEPAF.

## Fuentes de financiación

La presente investigación no ha recibido ayudas específicas provenientes de agencias del sector público, sector comercial o entidades sin ánimo de lucro.

El Estudio PEPAF se financió con una beca del Instituto de Salud Carlos III del Ministerio de Sanidad, y se cofinanció con una ayuda del ERDF de la Unión Europea (FIS PI02/0015; RETICS G03/170 y RD06/0018/0018; CAIBERCAI08/01/0065).

## Datos y código analítico

Están disponibles por petición razonada. Los datos son solo los correspondientes a este artículo y proceden del Estudio PEPAF. Las peticiones deberán dirigirse al autor de correspondencia.

## Autoría

Ricardo Ortega: concepción y diseño del estudio, análisis e interpretación de los datos, borrador del artículo y aprobación definitiva de la versión que se presenta.

Gonzalo Grandes: adquisición de datos, revisión crítica del contenido intelectual y aprobación definitiva de la versión que se presenta.

Sagrario Gómez-Cantarino: concepción y diseño del estudio, revisión crítica del contenido intelectual y aprobación definitiva de la versión que se presenta.

## Conflicto de intereses

Los autores no tienen ningún conflicto de intereses que declarar.

## Anexo 1 Miembros del Grupo PEPAF

Equipo Directivo: Unidad de Investigación en Atención Primaria de Bizkaia, Servicio Vasco de Salud-Osakidetza. Investigador principal: Gonzalo Grandes; co-investigadores: Jesús Torcal, Ricardo Ortega, Alvaro Sánchez, Kepa Lizarraga, Javier Serra, Imanol Montoya.

Servicio Vasco de Salud-Osakidetza: Centro de Salud de Basauri-Aríz: Angel Fernández, Victor Manuel López, Lourdes Marijuán, Begoña Etxeguren, Victor Landa, Jesús Martínez, M. Mercedes Díez, Juan Ramón Lejarza, Judith González. Centro de Salud de Galdakao: Vidal Salcedo, Idoia Ibáñez, Agurtzane Ortego, Pedro Iraguen, Pilar Echevarria, Virginia Villaverde. Centro de Salud de Algorta: Amaia Ecenarro, M. Teresa Uribe, Carmen Moral, Eguskiñe Iturregui, Ana Belén Fernández.

Servicio Andaluz de Salud: Centro de Salud de Camas, Sevilla: José María Páez, M. Ángeles Tarilonte, Concepción Molina, Vicente Rodríguez, Isabel Villafuente, Mercedes Álvarez.

Servicio de Salud de Islas Baleares: Centro de Salud de Dalt Sant Joan, Mahón: Andreu Estela, José María Coll, Àngels Llach, Josep M. Masuet, Ana Moll, Monica Pons.

Servicio Catalán de Salud: Centro de Salud de Serraparera, Barcelona: Bonaventura Bolibar, Agustí Guiu, Amadeu Díaz, Xavier Martínez, M. Dolores Hernández, José Ignacio Olivares, Francisco Hernansanz, Rita Ayala, Ana Cascos.

Servicio de Salud de Castilla-La Mancha: Centro de Salud de San Fernando Cuenca III, Cuenca: Vicente Martínez, M. del Carmen García, M. Ángeles Gabriel, M. Luscinda Velázquez, Natividad Ortega, M. Jesús Segura, Rodrigo Cerrillo, Patricia López.

Servicio de Salud de Castilla y León: Centro de Salud de Casa Barco, Valladolid: Carmen Fernández, Amparo Gómez, Miguel Ángel Díez, Ruperto Sanz, Luis Miguel Quintero, José Ignacio Recio. Centro de Salud La Alamedilla, Salamanca: Luis García, José Antonio Iglesias, Manuel Gómez, Emilio Ramos, Pilar Moreno, Yolanda Castaño, Nadia Carrillo.

Servicio Gallego de Salud: Centro de Salud de Sardoma, Vigo: Pilar Gayoso, Luciano Casariego, Manuel Domínguez, José Ramón Moliner, Fernando Lago, M. Concepción Cruces, Marisa Enríquez.

Servicio Madrileño de Salud: Centro de Guayaba, Madrid: Tomás Gómez, Javier Martínez, José Antonio Granados, M. Ángeles Fernández, M. Isabel Gutiérrez, Carlos San Andrés, Concepción Vargas-Machuca, Cristina Díaz. Lo conocido sobre el tema

• La obesidad es considerada una enfermedad crónica como la hipertensión, la diabetes o la dislipemia.
• Se produce por un balance energético positivo: la ingesta de calorías supera al gasto.
• Todas las intervenciones van dirigidas a reducir el peso o la grasa corporal pero no a «curarla».

Qué aporta este estudio

• La obesidad se puede «curar», a diferencia de las otras enfermedades crónicas.
• Es posible dejar de tener obesidad general (OG), obesidad abdominal (OA) y/o exceso de grasa corporal (EGC) a los 2 años.
• Entre los que normalizan su índice de masa corporal, la mitad siguen con OA o EGC, mientras que entre los que normalizan su perímetro abdominal, solo la quinta parte siguen con OA o EGC.
